# The Association of Quality of Life with Psychosocial Factors in Adolescents with Tourette Syndrome

**DOI:** 10.1007/s10578-023-01656-0

**Published:** 2024-02-04

**Authors:** Kelly H. Watson, Michelle Eckland, Jessica M. Schwartzman, Andrew Molnar, Whitney Boon, Matthew Hiller, Seth Scholer, Rachel Mace, Alice Rothman, Daniel O. Claassen, Heather R. Riordan, David A. Isaacs

**Affiliations:** 1 Department of Neurology, Vanderbilt University Medical Center, 1161 21St Avenue South, A-0118 MCN, Nashville, TN 37232 USA; 2 Department of Psychiatry and Behavioral Sciences, Vanderbilt University Medical Center, Nashville, TN USA; 3 Division of Developmental-Behavioral Pediatrics, Children’s Hospital Los Angeles, Los Angeles, CA USA; 4 Department of Pediatrics, Keck School of Medicine, University of Southern California, Los Angeles, CA USA; 5 Department of Pediatrics, Vanderbilt University Medical Center, Nashville, TN USA; 6 Phelps Center for Cerebral Palsy and Neurodevelopmental Medicine, Kennedy Krieger Institute, Baltimore, MD USA; 7 Department of Neurology, Johns Hopkins Hospital, Baltimore, MD USA

**Keywords:** Tourette syndrome, Tics, Adolescence, Quality of life, Family functioning

## Abstract

Individuals with Tourette syndrome (TS) have poorer quality of life (QoL) than their peers, yet factors contributing to poor QoL in this population remain unclear. Research to date has predominantly focused on the impact of tics and psychiatric symptoms on QoL in TS samples. The aim of this cross-sectional, multi-informant study was to identify psychosocial variables that may impact adolescent QoL in TS. Thirty-eight adolescents aged 13 to 17 with TS and 28 age-matched controls participated with a caregiver. No group differences were found on QoL, although the TS group reported reduced QoL compared to population normative data. In the TS group, reduced QoL was associated with lower self-esteem, poorer family functioning, higher stress, and greater depression and anxiety; QoL was unrelated to tic severity. In regression analyses, after adjusting for covariates, family functioning was the strongest predictor of QoL. These results emphasize the need to further explore the influence of psychosocial factors, particularly family functioning, on QoL in adolescents with TS.

**Supplementary Information:**

The online version contains supplementary material available at 10.1007/s10578-023-01656-0.

Tourette syndrome (TS) is a neurodevelopmental disorder characterized by a childhood-onset of involuntary motor and vocal tics that are often difficult to control and distressing to the individual. TS affects 0.3–1% of school-aged children worldwide [53]. Tics typically peak in severity by mid-adolescence and diminish during late adolescence, though tics persist into adulthood for more than 80% of individuals [47] and remain moderate-to-severe in adulthood for approximately 20% [4]. While tics are the hallmark feature of TS, 90% of patients are diagnosed with at least one psychiatric comorbidity, the most prevalent being attention-deficit/hyperactivity disorder (ADHD) and obsessive–compulsive disorder (OCD,[27]). Comorbid psychiatric symptoms follow a typical course, and while some symptoms (e.g., hyperactivity) generally improve during adolescence, many persist into adulthood [4, 27].

Many studies have demonstrated that children, adolescents, and adults with TS report lower quality of life (QoL) compared to their peers [6, 7, 13, 17, 18, 20, 61–63]. QoL refers to an individual’s evaluation of both the positive and negative aspects of their life circumstances in the context of their values and goals, and it is broadly a measure of subjective well-being [26]. QoL is increasingly recognized as an important endpoint in research and clinical practice for people with chronic disorders as this construct emphasizes the individual’s overall life satisfaction in the presence of disease [25]. QoL is also predictive of future health outcomes, including all-cause mortality [45].

The determinants of poor QoL in individuals with TS are likely multifactorial. For many patients, tics cause significant pain and discomfort, interfere with concentration in social and/or classroom settings, and contribute to stigmatization and difficulties in self- and peer-acceptance [10, 61–63, 72]. Despite this, evidence of the association between tic severity and QoL is mixed, with some studies observing reduced QoL in those with greater tic severity [13, 16, 61–63] but other studies failing to observe such a relationship [3, 17]. Notably, in studies that assess both tics and comorbid psychiatric symptoms, the latter often pose greater challenges to the individual and family QoL than tics themselves [16, 20]. Research has consistently demonstrated that psychiatric comorbidities have a marked adverse effect on QoL in TS [3, 6, 7, 16]. As such, national and international TS practice guidelines recommend routine evaluation and treatment of psychiatric comorbidities in individuals with TS [40, 46].

To date, most studies examining QoL determinants in TS have focused on tics and psychiatric symptoms, but several lines of evidence suggest additional psychosocial factors may also affect QoL in individuals with TS. First, psychological stress is elevated in TS [21], which is the perception of environmental demands straining adaptive ability [9]. Stress is known to exert a profound effect on QoL in individuals with chronic disease [52]. Second, there is a small body of evidence that individuals with TS report lower self-esteem than their peers (e.g., [67]). Self-esteem is an individual’s global appraisal of their worth as a person, and longitudinal studies have demonstrated its long-term health impact, as low self-esteem in adolescence has been found to be associated with higher levels of unemployment and greater physical and mental health problems in adulthood [56, 64]. Third, social difficulties are prevalent in TS, including challenges making friends, dating problems, difficulties interpreting social cues, and high levels of bullying [37, 61–63, 72]. Given extensive research establishing a robust link between social relationships and health [28], poor quality peer relationships may be an important determinant of QoL in TS. Lastly, families affected by TS report higher levels of family dysfunction (e.g., disruptions to family routines, communication, and cohesiveness; [68]). The family environment is an important context for physical and emotional support and security, and family dysfunction has been shown to be associated with reduced QoL in other clinical populations [66]. While heightened stress, low self-esteem, poor peer relationships, and family dysfunction are four recognized psychosocial challenges facing many individuals with TS, their unique contributions to QoL in TS remain largely unexplored.

The above psychosocial factors are of particular relevance in adolescence. Adolescence is the developmental period when tics and many other comorbid psychiatric symptoms of TS peak in severity [23, 54] and is characterized by considerable developmental transitions, heightened levels of stress and dynamic conceptions of self, peers, and family [71]. Further, identifying modifiable risk factors for poor QoL in adolescence provides an earlier window for intervention to improve outcomes.

The aims of the current multi-informant, cross-sectional study are to compare the QoL, self-esteem, perceived stress, perceived peer relationship quality, and perceived family dynamics between TS adolescents and age-matched controls and to examine the independent association of select psychosocial factors with adolescent QoL. First, we hypothesize significant between-group differences such that adolescents with TS will report lower QoL, greater stress, lower self-esteem, poorer peer relationship quality, and greater family dysfunction than age-matched controls. Second, we hypothesize that greater stress, lower self-esteem, lower quality of peer relationships, and an unsupportive family environment will each independently be associated with reduced QoL in TS adolescents, after accounting for tic and psychiatric symptom severity.

## Method

### Participants

Forty-six adolescents (ages 13–17) with tic disorders and 28 age-matched controls enrolled in the study. However, eight tic disorder participants had presentations consistent with functional tic disorder [22], (see Table S1 for participant characteristics), and their data were excluded from the analysis, resulting in a final dataset of 38 adolescents who met *Diagnostic and Statistical Manual of Mental Disorders, 5th edition* (DSM-5) criteria for TS and 28 controls (*N* = 66). All adolescents participated with an adult caregiver (see Table S2 for relationship details). Our cohort was predominantly White, non-Hispanic. The TS arm had a higher proportion of males (63.2%) than females (36.8%), while the sexes, as assigned at birth, were evenly represented in the control arm. Demographic characteristics are shown in Table 1.

**Table 1:** Demographic and clinical characteristics

Variable	Controls (n = 28)	TS^†^ (n = 38)	Pearson χ^2^ or Wilcoxon rank sum
Sex assigned at birth (M:F)	14:14	24:14	χ^2^(1) = 1.1
Gender (M:F)	15:13	24:14	χ^2^(1) = 0.6
Age (years)	15 (13–16)^‡^	15 (13–16)	z = − 0.4
Race			
Asian Native Hawaiian or Other Pacific Islander Black or African American White More than one race	1 0 2 22 3	0 1 1 33 3	χ^2^(4) = 3.1
Ethnicity			
Hispanic or Latino(a) Not Hispanic or Latino(a)	1 27	3 35	χ^2^(1) = 0.5
Comorbid disorders, previously diagnosed^§^			
Attention-deficit/hyperactivity disorder Obsessive–compulsive disorder Anxiety Depression Autism spectrum disorder None of above comorbid disorders	5 0 5 4 1 18	21 19 26 14 3 1	χ^2^(1) = 9.4** χ^2^(1) = 19.7*** χ^2^(1) = 16.6*** χ^2^(1) = 4.1* χ^2^(1) = 0.5 χ^2^(1) = 29.9***
Age of tic onset (years)	–	5.5 (4–7.5)^‡^	–

^*^p < 0.05; **p < 0.01; ***p < 0.001

^†^All tic disorder participants met DSM-5 criteria for Tourette syndrome (TS)

^‡^Median (interquartile range)

^§^Some participants had received multiple comorbid psychiatric diagnoses

### Study Procedures

The study protocol and outcomes were pre-registered on clinicaltrials.gov (NCT04449003). Adolescents with TS were recruited from the Vanderbilt Center for Tourette Syndrome and Other Tic Disorders. Controls were recruited from Vanderbilt Pediatric Clinics (24%), the Vanderbilt Research Notifications Distribution List (16%), and by word of mouth (60%). Recruitment and enrollment occurred from March 2021 through December 2022. Inclusion criteria for the TS arm included: 13–17 years of age, diagnosis of TS or other chronic tic disorder per DSM-5 criteria, English proficiency (as most study measures were available only in English), and participation of an adult caregiver with English proficiency. Exclusion criteria for the TS arm included diagnosis of a severe medical condition (e.g., heart transplant) and cognitive or behavioral impairment (e.g., intellectual disability) precluding ability to complete self-report questionnaires. Inclusion and exclusion criteria for controls were identical to the TS arm with the exception that controls were excluded if they had any history of tics.

All study procedures occurred during a single visit. A movement disorders neurologist with expertise in TS (HR, DI) interviewed all TS participants, with their caregiver, to confirm the diagnosis. Control participants were not interviewed. Adolescents and caregivers completed separate paper-based questionnaires concurrently in the same room. A research coordinator was in the room to address questions, minimize adolescent-caregiver communication, and verify completeness of the scales. TS and control participants completed the same questionnaires. Total time ranged from 60 to 90 min for TS participants and 30–45 min for controls.

### Measures

*Tic severity and impairment.* The Yale Global Tic Severity Scale (YGTSS; [32, 35]) is the gold-standard, semi-structured interview for quantifying tic severity and impairment. YGTSS total tic scores and impairment scores each range from 0 to 50, with higher scores indicating greater tic severity and impairment, respectively.

*Quality of life.* Adolescents completed the Youth Quality of Life Research Version (YQOL-R; [44]) to assess QoL. The self-report questionnaire includes 42 items about multiple domains, including sense of self, relationships, and environment. The total score was used for analyses, with higher scores indicating better QoL.

*Self-esteem.* Adolescents completed the 10-item Self Esteem Scale [51]. Items are rated on a 4-point Likert scale with higher scores indicating higher self-esteem.

*Stress.* Adolescents completed the Perceived Stress Scale (PSS; [31]), a 10-item measure assessing individuals’ appraisal of life stress. Scores range from 0 to 40, with higher scores indicating greater stress. The PSS is a widely used measure that has shown good psychometric properties in adolescence [31]. The 30-item Daily Life Stressors Scale was administered to assess the perceived burden from daily stressors (DLSS; [30]). Scores range from 0 to 120, with higher scores indicating greater stress burden.

*Peer relationship quality.* Adolescents and caregivers completed the PROMIS Pediatric Peer Relationships Short Form (PROMIS Peer; [14]) to assess peer relationship quality over the previous 7 days. The adolescent version has 8 items while the caregiver proxy version has 7 items. Items are rated on an 5-point Likert scale (1 = Never to 5 = Always). Higher scores are indicative of better friendship quality and peer acceptance.

*Family functioning.* Adolescents and caregivers completed the McMaster Family Assessment Device (FAD; [38]), a 60-item measure that yields scores for seven scales about family functioning: problem solving, communication, affective responsiveness, roles, affective involvement, behavior control, and general functioning. Individual scale items are rated on a 4-point Likert scale based on how well each statement describes the family.

*Family impact.* Caregivers completed the 36-item PedsQL-Family Impact Module (PedsQL Family; [65]). The measure includes the following scales: caregiver physical functioning, emotional functioning, social functioning, cognitive functioning, worry, communication, family daily activities, and family relationships. The Total Score was used for analyses, with higher scores indicating less impact of the child’s health on family functioning.

*Anxiety and depression symptoms.* Adolescents and caregivers completed the widely used, 47-item Revised Children’s Anxiety and Depression Scale (RCADS; [8]). Items are rated on a 4-point Likert scale (0 = Never to 3 = Always). The RCADS assesses five anxiety subtypes and depression symptoms. The anxiety and depression total symptom score was used in the present analyses, with higher scores indicating greater severity of symptoms.

*ADHD and related symptoms.* Caregivers completed the 45-item Conners 3 Parent Short Form (Conners 3-S; [11]) to assess symptoms of ADHD and associated behavioral difficulties. Conners 3 is widely used in research and clinical practice. Raw scores and *T* scores are obtained on six scales: Hyperactivity/Impulsivity, Inattention, Learning Problems, Executive Functioning, Aggression, and Peer Relations. Higher scores indicate greater parental concerns in these domains. In addition, two validity indices are calculated: Negative and Positive Impression.

*Co-occurring psychiatric disorders.* Caregivers completed a questionnaire requesting details of their adolescents’ prior psychiatric diagnoses and treatments. Comorbid neurologic and psychiatric disorders were not verified by clinical interview.

### Statistics

Statistical analyses were conducted with STATA 17.0. We examined missingness at the individual item level for all questionnaires. Questionnaire data were 99.97% complete. Given the small fraction of missing data at the individual item level for all questionnaires, we imputed missing item values with the mean of all non-missing values for that item across all participants, i.e., with mean substitution. Total scale and subscales scores were calculated following this imputation procedure. Following this imputation procedure, additional imputation was performed for the Total Tic Score since YGTSS paper records were misplaced for three TS participants (YGTSS data 92.1% complete). Total Tic Score was imputed using multivariate imputation with Markov Chain Monte Carlo procedures. The following variables were included in the imputation model: sex, Self-Esteem scale score, PSS score, DLSS score, adolescent- and caregiver-reported PROMIS Peer scores, all adolescent- and caregiver-reported FAD scale scores, adolescent- and caregiver-reported RCADS Total Anxiety and Depression raw scores, all Conners 3-S subscale raw scores, YQOL-R total score, and PedsQL Family total score. This set of variables exhibited a joint normal distribution among TS participants [per Henze–Zirkler method: χ^2^(1) = 2.7, *p* = 0.10; per Doornik–Hansen method: χ^2^(66) = 81.5; *p* = 0.10]. We created a total of 20 datasets with imputed YGTSS Total Tic Score, which were only used in the regression analysis. The non-imputed Total Tic Score was used for the correlation analysis.

Following imputation procedures, measures of central tendency were calculated for all variables. To determine internal consistency of adolescent- and caregiver-reported scales, we computed both Cronbach’s α and McDonald’s ω ([15],Table S3).

To compare demographic variables and scale scores between TS and control participants, we used Pearson’s χ^2^ test for categorical variables and Wilcoxon’s rank-sum test for continuous variables. Given concerns that the study control population was not representative of a community adolescent sample, we also compared TS and control participant group scores to normative data or clinical cut-offs, when available, using a one-sample Wilcoxon’s signed-rank test. Normative data and clinical cut-offs were obtained from scale user manuals and/or peer-reviewed journal publications (see references in Table S5).

To examine the interrelationship between scales, we calculated Spearman’s rank correlation coefficients (*r*_*s*_), stratified by TS status. When relevant, we used raw or scaled scores, rather than T-scores, in the correlation analysis since T-scores incorporate information from normative populations that are irrelevant to within-group analyses. As noted above, we used the non-imputed YGTSS Total Tic Score for the TS group correlations. We applied Benjamini and Yekutieli [2]’s method to correct for multiple comparisons in the correlation analysis.

**Fig. 1:**
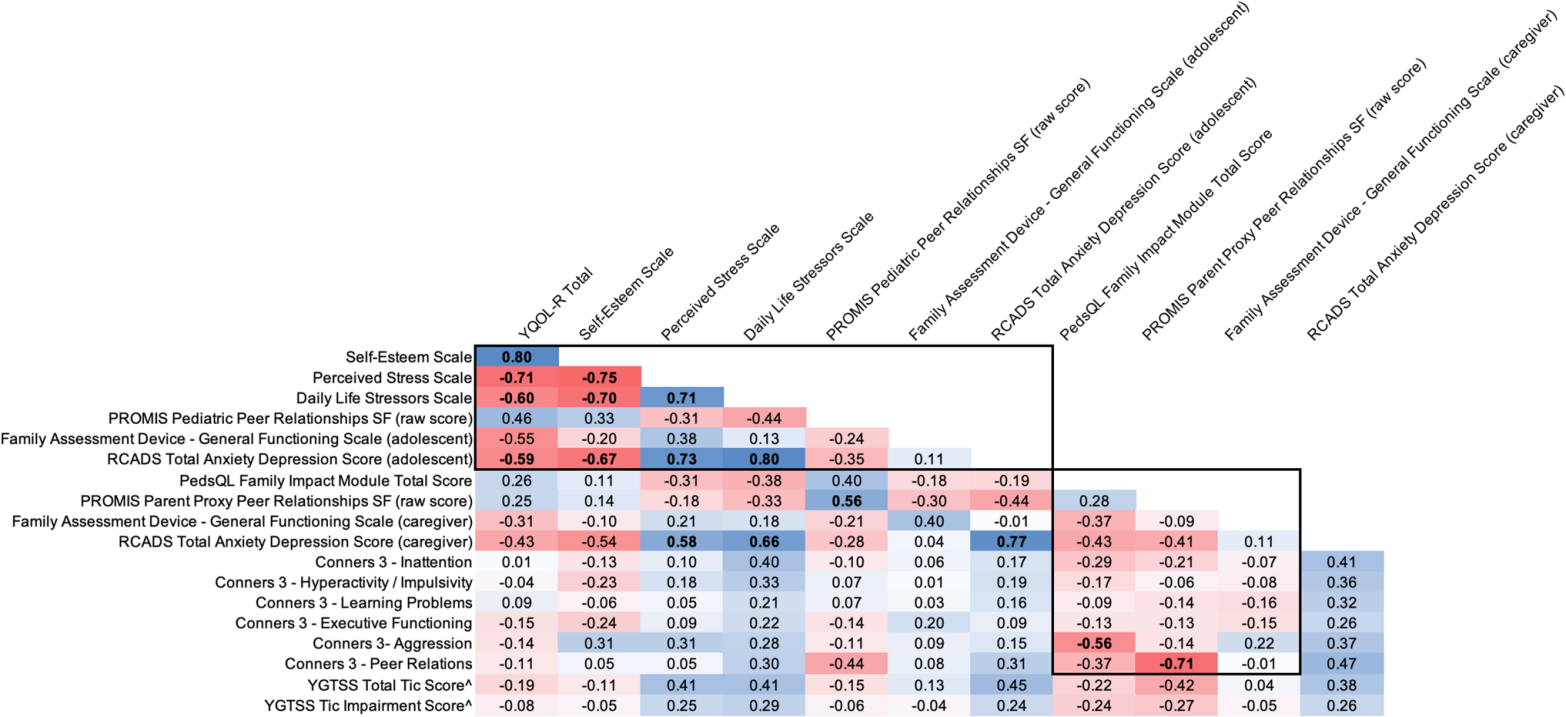
The bold-outlined section in the upper left quadrant of the matrix contains correlations between adolescent-reported measures. The bold-outlined section in the lower right quadrant of the matrix contains correlations between caregiver-reported measures. Red and blue shading signify negative and positive correlations, respectively, while intensity of shading signifies the strength of the correlation. Bolded values indicate statistically significant correlation following correction for multiple comparisons; given 143 statistical comparisons in the above matrix, any correlation with magnitude less than 0.56 (associated p > 0.00039) is not significant. ^YGTSS Total Tic Score and Tic Impairment Score were available for 35 TS participants; these scores were missing for 3 TS participants

To further examine the relationship of QoL with psychosocial factors, we conducted a least absolute shrinkage and selection operator (LASSO) regression for inference using cross-fit partialing out (double machine learning) with plugins method and ten folds per cross-fit. Separate LASSO regression analyses were performed for TS and control participants. In the LASSO regression analysis for TS participants, YQOL-R total score served as the dependent variable; DLSS score, adolescent-reported PROMIS Peer score, and adolescent-reported FAD General Family Functioning score served as independent variables of interest; and the following served as control variables: age, sex, YGTSS Total Tic Score, Self-Esteem Scale score, PSS score, adolescent-reported RCADS Total Anxiety-Depression raw score, and Conners 3-S raw scores for all scales. Because of the high collinearity between the DLSS and Self-Esteem Scale scores (*r*_*s*_ =  − 0.70; Fig. 1), we opted to include the Self-Esteem Scale score as a covariate rather than an independent variable of interest in the model. LASSO regression was performed iteratively for each of the 20 datasets with imputed YGTSS Total Tic Score values. Model and coefficient characteristics are reported for each of these 20 LASSO regressions. In the LASSO regression analysis for controls, the same variables were included in the model with the exception that YGTSS Total Tic Score was not included as a control variable. Since YGTSS Total Tic Score was the only variable imputed with multivariable imputation and since the control regression model did not contain this variable, the LASSO regression was conducted only once for controls.

## Results

### Clinical Characteristics of the Samples

Among TS participants, the prevalence of ADHD (55.3%), OCD (50.0%), depression (36.8%), and autism spectrum disorder (7.9%), per caregiver-report of previous diagnoses, was consistent with other TS cohorts, while the prevalence of anxiety (68.4%) was elevated [5, 27]. Almost all (97.4%) TS participants had at least one co-occurring diagnosis, and 68.4% had two or more of the co-occurring diagnoses listed in Table 1. Age of reported tic onset and tic severity [32, 57] were typical for a TS sample population recruited from a tertiary care center.

### Between-Group Contrasts and Comparisons to Normative Samples

TS and control groups did not significantly differ on the YQOL-R total or subscale scores, Self-Esteem Scale score, adolescent- and caregiver-reported PROMIS Peer scores, or scores on the FAD (Table 2). Significant between-group differences were evident for the PSS score, DLSS score, adolescent- and caregiver-reported RCADS Total Anxiety Depression scores; Inattention, Hyperactivity/Impulsivity, and Learning Problems subscale scores of the Conners 3-S, and PedsQL Family score. Details on between-group contrasts of subscale scores on select measures are shown in Table S4.

**Table 2:** Between-group contrasts of rating scale scores

Scale	Controls (n = 28)	TS (n = 38)	Wilcoxon rank sum test statistic
Adolescent-Report Scales			
Youth Quality of Life-Research Version (YQOL-R) Total	82.0^‡^ (71.4–90.1)	75.9 (63.2–90.3)	z = 0.7
Self-Esteem Scale	29.5 (26.5–34)	28.5 (25–34)	z = 0.4
Perceived Stress Scale	15 (9–18.5)	18.5 (11–25)	z = − 2.1*
Daily Life Stressors Scale	27.5 (18–37)	37 (27–51)	z = − 2.2*
PROMIS Pediatric Peer Relationships Short Form 8a (T-score)	42.6 (38.8–47.4)	43.7 (38.8–50.9)	z = − 0.3
Family Assessment Device, General Functioning Scale	1.8 (1.5–2.3)	2.0 (1.5–2.5)	z = − 0.8
Revised Children’s Anxiety and Depression Scale, Total Anxiety Depression T-score	46 (38–53)	57 (45–64)	z = − 2.7**
Caregiver-Report Scales			
PedsQL Family Impact Module Total	95 (67–99)	73 (53–78)	z = 3.9***
PROMIS Parent Proxy Peer Relationships Short Form 7a (T-score)	45 (42–51)	42.5 (36–51)	z = 1.6
Family Assessment Device, General Functioning Scale	1.6 (1.4–2.0)	1.7 (1.3–2.0)	z = 0.0
Revised Children’s Anxiety and Depression Scale, Total Anxiety Depression T-score	53 (45–61)	63 (55–70)	z = − 3.0**
Conners-3 Parent Short Form (T-scores)			
Inattention Hyperactivity/Impulsivity Learning Problems Executive Functioning Aggression Peer Relations	57 (49–69) 57 (48–64) 49 (46–54) 53 (47–62) 49 (45–52) 53 (46–68)	71 (62–81) 81 (63–90) 57 (50–66) 61 (49–71) 51 (45–60) 59 (45–90)	z = − 3.2** z = − 4.3*** z = − 2.6** z = − 1.9 z = − 1.2 z = − 1.4
Clinician-Administered Scales			
Yale Global Tic Severity Scale, Total Tic Score	–	29^†^ (18–35)	–
Yale Global Tic Severity Scale, Tic Impairment Score	–	20^†^ (10–30)	–

Additional between-group contrast scale and subscale score information is available in Table S4

^*^p < 0.05, **p < 0.01, ***p < 0.001

^‡^Median (interquartile range)

^†^For 35 participants; 3 participants were missing YGTSS Total Tic Score and Tic Impairment Score

Full details of the comparison of TS and control groups to population norms (for select scales where such data were available) are presented in Tables S5 and S6. Notable findings are reported here. TS group scores were significantly lower than normative population scores for YQOL-R and both adolescent- and caregiver-reported PROMIS Peer scores [57]. TS group scores were significantly higher than normative scores on multiple subscales from the RCADS and Conners 3-S, indicating the TS group experiences more symptoms of anxiety and depression and more hyperactivity/impulsivity, inattention, learning difficulties, executive dysfunction, and peer relation difficulties than normative populations. TS adolescent-reported FAD—Affective Involvement scores were significantly higher than the established clinical threshold, indicating group-level dysfunction in that domain. Control group scores were significantly lower than normative population scores for adolescent- and caregiver-reported PROMIS Peer scores, indicating the control group experiences lower peer relationship quality than normative populations. Control group scores were significantly higher than normative population scores on multiple Conners 3-S scales, indicating the control group experiences more inattention, hyperactivity/impulsivity, and peer relations difficulties than normative populations.

### Correlations

Among TS participants, scores from the PSS and DLSS strongly correlated (*r*_*s*_ = 0.71; Fig. 1). Both stress scores strongly correlated with YQOL-R total score (*r*_*s*_ =  − 0.71 and − 0.60, respectively), Self-Esteem Scale score (*r*_*s*_ =  − 0.75; − 0.70), and RCADS Total Anxiety and Depression score (*r*_*s*_ = 0.73; 0.80), indicating greater perceived stress is associated with lower QoL, lower self-esteem, and more severe anxiety and depression symptoms. Among TS participants, YQOL-R total score most strongly correlated with the following, in order of strongest to weakest correlation magnitude: Self-Esteem Scale score, PSS score, DLSS score, and RCADS Total Anxiety Depression score (Fig. 1; Fig. S1). The degree of correlation of YQOL-R total score with adolescent-reported FAD General Functioning score and adolescent-reported PROMIS Peer score was moderate (*r*_*s*_ =  − 0.55 and 0.46, respectively) but did not meet the significance threshold after correction for multiple comparisons. The only measure that significantly correlated with PedsQL Family score was the Aggression subscale score from the Conners 3-S. PedsQL Family and YQOL-R total scores did not significantly correlate. Notably, PSS and DLSS scores correlated with YGTSS Total Tic Score (*r*_*s*_ = 0.41 for both correlations), indicating greater stress is associated with greater tic severity; however, the strength of these correlations did not meet statistical significance following correction for multiple comparisons. Neither YQOL-R total score nor PedsQL Family score significantly correlated with YGTSS Total Tic or Tic Impairment Scores. See Fig. S2 for the control sample correlation matrix.

### LASSO Regression

Across all twenty datasets with imputed YGTSS Total Tic Scores for TS participants, the LASSO regression models explained a significant amount of variance in YQOL-R [χ^2^(3) ranged from 25.3 to 27.3; p < 0.0001 for all models]. Of the three independent variables of interest, only adolescent-reported FAD General Functioning score was significantly associated with YQOL-R total score (p < 0.001) across all imputed datasets. Per the regression models, each 0.1-point increase in FAD General Functioning score was associated with a 0.85-point decrease in YQOL-R total score, indicating unhealthy family functioning is associated with lower adolescent QoL, after adjusting for perceived daily life stressor burden, peer relationship quality, and a subset of control variables. The DLSS score and adolescent-reported PROMIS Peer scores were not significantly associated with YQOL-R total score in the LASSO regression models. Results of the LASSO regression for control participants yielded similar findings, with FAD General Functioning score (p < 0.001), but not DLSS or adolescent-reported PROMIS Peer scores, significantly associated with YQOL-R total score. Additional details of the regression output for all participants are available in the Supplemental Material (Tables S7, S8).

## Discussion

The objectives of the present study were to compare the QoL of adolescents with TS to age-matched controls and to examine the association of psychosocial factors with QoL in adolescents with TS. The present study identified five main findings. First, adolescents with TS reported similar QoL as age-matched controls but reduced QoL compared to normative populations. Second, adolescents with TS reported higher stress than age-matched controls. Third, caregivers of adolescents with TS reported worse functioning because of their child’s health than caregivers of age-matched controls. Fourth, among adolescents with TS, higher levels of stress, lower self-esteem, and poorer family functioning were negatively correlated with QoL. Lastly, family functioning, but not stress nor quality of peer relations, predicted QoL in TS after adjusting for covariates. Each of these findings will be discussed in turn.

QoL did not significantly differ between adolescents with TS and recruited controls. This finding seems contrary to most previous research, which has documented reduced QoL in TS from childhood through adulthood (e.g., [6, 7, 20]. It is noteworthy that many prior studies assessing QoL in TS focused on health-related QoL, as assessed by the disorder-specific Gilles de la Tourette Syndrome—Quality of Life Scale [6, 7, 55]. This measure is informative for understanding the impact of characteristic TS symptoms on QoL. In the present study, however, we used a generic QoL measure for two reasons: first, it captures a broader QoL construct that is more informative for clarifying the association between QoL and characteristics not classically considered part of the core TS phenotype (e.g., social interaction difficulties); second, it allows direct QoL comparisons of individuals with TS to their peers. In the present study, despite the lack of difference in YQOL-R scores between TS and control groups, adolescents with TS reported significantly reduced QoL compared to established YQOL-R population norms, which suggests adolescents with TS experience reduced QoL in non-disorder-specific domains. It is likely that our recruited control sample was not representative of healthy adolescents since they reported poorer peer relationships and family functioning as well as elevated symptoms of anxiety, depression, and ADHD compared to normative data. This may have been because recruitment occurred during the COVID-19 pandemic, which had a significant impact on daily life across the world [48]. Additionally, many control participants were recruited from academic medical center channels, and families may have been interested in participating in research on psychological heath and QoL because their adolescent was currently experiencing challenges. Taken together, the present findings underscore the value of using generic in addition to disorder-specific QoL measures in TS samples and the importance of recruiting a control sample to best understand QoL in this vulnerable population.

In the present study, tic severity and impairment were unrelated to adolescent self-report of their QoL. Findings on the association between tic severity and QoL have been inconsistent [3, 13, 16, 17, 61, 61, 62, 62, 63, 63], likely in part due to between-study differences in sample population characteristics, measures administered, and analytic approach. Notably, significant associations between tic severity and QoL have often been found in studies relying on self-report measures of tic severity [13], an association which could partially be accounted for by shared method variance. In contrast, several studies that have used a multi-informant approach, obtaining self-reports of QoL and clinician ratings of tic severity, have reported a non-significant association between these domains [3, 55]. Our study results suggest tic severity does not account for variability in adolescent QoL. Given tic reduction is often a primary treatment focus, these findings highlight the clinical relevance of examining factors beyond tic severity that may contribute to poor adolescent QoL in TS.

In the present sample, 97% of adolescents with TS had received a prior diagnosis of at least one co-occurring psychiatric condition, with the most prevalent being anxiety, ADHD, and OCD. This clinical presentation is consistent with the broader literature that has documented up to 90% of individuals with TS have at least one psychiatric comorbidity [49]. Furthermore, we found that anxiety and depression symptoms were significantly and negatively associated with adolescent QoL. Notably, caregiver-reported ADHD symptoms were unrelated to adolescent-reported QoL. While surprising, this finding is consistent with at least two prior studies that found limited evidence for the association between QoL and ADHD symptoms in TS using a multi-informant approach [17, 19]. Overall, our results align with the well-established finding that psychiatric symptoms are associated with poor QoL in TS. Yet, current study results reveal additional psychosocial factors may account for variability in QoL in TS, and we will now highlight the major findings regarding the four psychosocial factors we examined: stress, self-esteem, peer relationships, and family functioning.

Adolescents with TS reported significantly higher perceived stress than age-matched controls. While not significant after correction for multiple comparisons, perceived stress in adolescents with TS was correlated with tic severity such that greater stress was associated with more severe tics. The relationship between stress and tics is complicated and likely bidirectional. On the one hand, tics can interfere with daily functioning, by, for example, impairing focus in the classroom or in conversation, eliciting unwanted attention from others, or causing pain or discomfort, which may cause heightened stress [10, 43, 59, 61–63]. On the other hand, individuals with TS are known to be sensitive to stress [21], and research has shown that psychosocial stress can exacerbate the frequency and intensity of tics [33]. In the present study, greater stress was negatively associated with adolescent QoL; however, stress was not significantly associated with QoL after adjusting for other psychosocial factors in the regression analysis. Research on the longitudinal course of stress in TS, as well as its causes and effects, is warranted given the widespread, deleterious impact of chronic stress on health and QoL [36].

Self-esteem of adolescents with TS did not significantly differ from that of age-matched controls or normative populations. Limited research has compared self-esteem levels in TS to healthy controls, and the findings from the few studies have been inconsistent (e.g., [60, 67]. Although TS can be a stigmatizing disorder, these findings suggest that, as a group, adolescents with TS do not report more negative feelings about themselves compared to their peers without tics. This is especially noteworthy given the high prevalence of psychiatric disorders in the present sample and because adolescence is marked by a strong desire for peer belonging [41]. Across both adolescents with TS and controls in our study, self-esteem was the variable that most strongly correlated with QoL. Future research involving longitudinal assessments is needed to determine if low self-esteem is a risk factor for poor QoL in adolescents with TS. Crucially, research has shown self-esteem can be improved through behavioral interventions [24].

The quality of peer relationships, based on adolescent and caregiver reports, did not significantly differ between adolescents with TS and controls. This was unexpected given prior research has documented widespread social problems in TS, including reduced peer acceptance, withdrawn and aggressive behaviors, and social deviance [16, 60]. However, because *both* the TS and control samples reported lower quality peer relations compared to the PROMIS Peer scale normative population, our findings suggest adolescents with TS do experience peer relational problems, and, as discussed above, our control sample may not be representative of healthy adolescents. While peer relationship quality correlated with QoL in our TS sample, the significance of this association did not survive correction for multiple comparisons. Additionally, peer relationship quality was not significantly associated with TS adolescent QoL in the regression model after accounting for other symptoms and psychosocial factors. This was unexpected based on prior research in other populations showing that peer relationships are important for QoL, especially in adolescence [1]. Given the small sample size, these findings are preliminary and warrant further research in larger samples.

Relative to caregivers of control participants, caregivers of adolescents with TS reported a greater impact on their family from their child’s health. Among adolescents with TS, extent of family impact correlated only with severity of aggression, not with QoL, tic severity, or other psychiatric symptoms. Results suggest that aggression in adolescents with TS is a risk factor for caregiver strain and an intervention target for families. These findings align with prior studies showing co-occurring behavioral difficulties account for much of the disorder’s impact on the family, including higher levels of parental stress, reduced parental self-perceived competence, and greater caregiver burden [12, 50, 58]. There were no group differences in adolescent- or caregiver- reported family functioning on the FAD. However, reports from adolescents with TS exceeded the clinical cut-off on the Affective Involvement scale of the FAD, suggesting TS adolescents viewed their family as overly involved and protective. Greater familial affective involvement can have adverse consequences for youth, especially in adolescence when it is developmentally appropriate to seek greater autonomy and learn skills to independently cope with challenges [71].

Consistent with our hypothesis, adolescent-reported family functioning was significantly and positively correlated with QoL for both the TS and controls groups, although the strength of the correlation was not significant after correction for multiple comparisons. However, in the regression analysis for both TS and control participants, adolescent-reported family functioning was the *only* significant predictor of QoL after accounting for psychiatric symptoms and other psychosocial factors. These findings are consistent with a small body of research on the role of family functioning in the QoL of adolescents with TS and suggest dysfunctional family dynamics may play a greater role in the QoL of adolescents with TS than psychosocial stress or peers [19]. Due to the high heritability of TS and its co-occurring disorders [42], many adolescents with TS may live with a parent or other family member affected by a psychiatric disorder [70], and a large body of evidence has linked parental psychopathology to greater negative parenting behaviors (e.g., [34]. Although we did not directly assess parenting behaviors in the present study, negative parenting practices (e.g., coercion, intrusiveness) may have contributed to greater family dysfunction [69] and poorer adolescent QoL. Given the pivotal influence of the family environment on adolescent development [39], and the malleability of parenting behaviors, findings highlight the importance of future research better understanding the specific family functioning factors that contribute to adolescent well-being in TS. Notably, caregiver-reported family functioning was not correlated with QoL for TS or control participants. The stronger association between adolescent-reported family functioning and QoL may partially be accounted for by shared method variance (i.e., self-reports). While the correlation between adolescent- and caregiver-reported family functioning was not statistically significant following correction for multiple comparisons, the correlation magnitude suggests there are meaningful similarities and differences in the perceptions of adolescents and caregivers. The study results highlight the value of assessing the adolescent viewpoint as part of research and clinical practice.

Findings from the current study should be interpreted in the context of several limitations. First, the cross-sectional study design precludes conclusions about the directionality of effects. A longitudinal study design with repeated assessments will permit stronger inferences on the role of psychosocial factors on adolescent well-being in TS. Second, study enrollment predominantly occurred during the height of the COVID-19 pandemic, which may have drastically influenced daily routines, peer interactions, and family relations for many study participants. TS and control participants may have been differentially affected by disruptions from COVID-19 in daily living. To partially address this potential confound, we compared both TS and control participant scores on study measures to representative normative data, when available. Third, the sample size was modest, which limited our statistical power. Fourth, as discussed above, the control sample may not have been representative of healthy adolescents since they were rated as having greater psychiatric symptoms and relational problems than normative samples. This may have been partly due to the second limitation (i.e., COVID-19 pandemic) and/or recruitment of many controls from an academic medical center. Finally, the FAD measure does not account for multiple parental households, most relevant for adolescents whose parents are separated. Study limitations were offset in part by several strengths. First, there was minimal missing data. Second, adolescents and caregivers completed the questionnaires during an in-person visit, which ensured that dyads completed them separately. Third, the study design included multi-informant ratings. Further, the present study obtained self-reports of adolescent QoL, which is an advantage given prior research demonstrating discrepant reports between parents and adolescents with TS, particularly in late adolescence [6, 7, 19, 55]. Fourth, most measures administered had representative normative data with which to compare the TS sample. Lastly, we examined the association of QoL in adolescents with TS with several potentially modifiable psychosocial factors.

## Summary

In summary, t﻿he findings of the present study demonstrate that while adolescents with TS report similar QoL to a recruited control sample, they report reduced QoL compared to normative populations. Furthermore, findings indicate adolescents with TS perceive higher levels of stress than their peers and caregivers of adolescents with TS report worse functioning than their peers. Importantly, adolescent tic severity was not related to QoL, suggesting that families and clinicians should not assume that tics interfere with adolescent well-being. Worse QoL in adolescents with TS was associated with more severe depression and anxiety, heightened stress, worse self-esteem, and family dysfunction. While the association between QoL and quality of peer relationships did not reach statistical significance, there was a numerical trend in the expected direction. The results of the present study emphasize the salience of families to QoL in TS adolescents. Most treatments for TS focus on the individual, but current findings suggest it may be beneficial for clinicians to systematically screen for family functioning, specifically assessing the perceptions of the adolescent, and offer family-focused psychosocial interventions to improve the QoL of adolescents with TS. Future research should involve longitudinal assessment of adolescents with TS, examining specific characteristics of the family environment (e.g., parenting behaviors, conflict) that may contribute to adolescent QoL, which ultimately could inform the development of targeted psychosocial family interventions. Future research in TS should also consider the potential roles of other modifiable psychosocial factors that were not examined in the present study but have been found to be associated with QoL in other clinical populations, such as perceived stigma and adolescent coping behaviors [29]. Identifying the risk factors for and protective factors against poor QoL in adolescents with TS is essential to informing intervention and improving care for this vulnerable population.

## Supplementary Information

Below is the link to the electronic supplementary material.

Supplementary file1 (DOCX 683 kb)

## Data Availability

The data that support the findings of this study are available from the corresponding author upon reasonable request.
