# Supplementary material for: The Association of Quality of Life with Psychosocial Factors in Adolescents with Tourette Syndrome
Source: Child Psychiatry Hum Dev. 2024 Feb 4;56(6):1586–97. doi: 10.1007/s10578-023-01656-0 (PMC11661522; doi:10.1007/s10578-023-01656-0)
Supplement: Supplementary file 1 — Supplementary file1 (DOCX 683 kb) [file 10578_2023_1656_MOESM1_ESM.docx]

**SUPPLEMENTAL MATERIAL**

**Table S1**

*Demographics and Clinical Characteristics of Functional Tic Disorder Participants*

| **Variable** |  |
| --- | --- |
| Sex Assigned at Birth (M:F) | 0 : 8 |
| Gender (M:F) | 0 : 8 |
| Age | 14.5 (14-15.5) |
| Race  Asian  Native Hawaiian or Other Pacific Islander  Black or African American  White  More than one race | 0  0  0  7  1 |
| Ethnicity  Hispanic or Latino(a)  Not Hispanic or Latino(a) | 1  7 |
| Comorbid Diagnosis  Attention-deficit/hyperactivity disorder  Obsessive compulsive disorder  Anxiety  Depression  Autism spectrum disorder | 3  3  4  4  0 |
| Age of onset of tic-like behaviors | 10 (7-13.5) |

**Table S2**

*Characteristics of Caregiver Relationship to Adolescent*

|  | **Controls** | **TS** |
| --- | --- | --- |
| Relationship to Adolescent^†^ |  |  |
| Mother | 20 | 33 |
| Father | 5 | 2 |
| Other Relative | 2 | 3 |
| Frequency of Contact |  |  |
| Every day | 27 | 36 |
| Several times per week but not every day | 1 | 2 |
| Once per week | 0 | 0 |
| Less than once per week | 0 | 0 |

*Note.* † missing relationship data for one of the controls

**Table S3**

*Internal Reliability of Self-Report Scales Across All Study Participants*

| **Scale** | **Cronbach’s α** | **McDonald’s 𝛚** |
| --- | --- | --- |
| **Adolescent-Report Scales** |  |  |
| Youth Quality of Life – Research Version |  |  |
| Self | 0.91 | 0.91 |
| Relationship | 0.89 | 0.89 |
| Environment | 0.85 | 0.86 |
| General Quality of Life | 0.90 | 0.92 |
| Total | 0.94 | 0.96 |
| Self-Esteem Scale | 0.88 | 0.88 |
| Perceived Stress Scale | 0.89 | 0.89 |
| Daily Life Stressors Scale | 0.85 | 0.86 |
| PROMIS Pediatric Peer Relationships Short Form 8a (raw score) | 0.89 | 0.87 |
| Family Assessment Device  Problem Solving  Communication  Roles  Affective Responsiveness  Affective Involvement  Behavior Control  General Functioning | 0.78  0.80  0.71  0.81  0.80  0.71  0.94 | 0.80  0.81  0.72  0.81  0.81  0.72  0.94 |
| **Caregiver-Report Scales** |  |  |
| PedsQL Family Impact Module  Physical Functioning  Emotional Functioning  Social Functioning  Cognitive Functioning  Communication  Worry  Daily Activities  Family Relationships  Total | 0.92  0.90  0.91  0.95  0.89  0.90  0.91  0.95  0.97 | 0.93  0.90  0.92  0.95  0.90  0.91  0.92  0.95  0.97 |
| PROMIS Parent Proxy Peer Relationships Short Form 7a (raw score) | 0.95 | 0.95 |
| Family Assessment Device  Problem Solving  Communication  Roles  Affective Responsiveness  Affective Involvement  Behavior Control  General Functioning | 0.80  0.80  0.71  0.79  0.65  0.70  0.91 | 0.81  0.81  0.69  0.82  0.69  0.72  0.91 |
| **Scale** | **Cronbach’s α** | **McDonald’s 𝛚** |
| Conners-3 Parent Short QuikScore Form (raw scores)  Inattention  Hyperactivity / Impulsivity  Learning Problems  Executive Functioning  Aggression  Peer Relations  Positive Interpretations  Negative Interpretations | 0.92  0.89  0.79  0.88  0.82  0.87  0.68  0.73 | 0.92  0.89  0.79  0.88  0.90  0.88  0.70  0.74 |

**Table S4**

*Between-Group Contrasts of Scores from Multidimensional Rating Scales*

| **Scale** | **Controls**  (n=28) | **TS**  (n=38) | **Wilcoxon Rank Sum Test Statistic** |
| --- | --- | --- | --- |
| **Adolescent-Report Scales**  Youth Quality of Life – Research Version  Sense of Self  Social Relationships  Environment  General Quality of Life  Total | 77.9^‡^ (63.2-86.4)  82.5 (73.6-88.6)  85.5 (77.5-91.5)  85.0 (68.3-95.0)  82.0 (71.4-90.1) | 72.5 (55.7-81.4)  78.9 (68.6-90.7)  86.0 (71.0-93.0)  83.3 (60.0-100.0)  75.9 (63.2-90.3) | z = 1.6  z = 0.4  z = 0.1  z = 0.1  z = 0.7 |
| Family Assessment Device  Problem Solving  Communication  Roles  Affective Responsiveness  Affective Involvement  Behavior Control  General Functioning  Revised Children’s Anxiety and Depression Scale (T-score)  Total Anxiety Depression  Total Anxiety  Total Depression  Obsessions-Compulsions  Social Phobia  Panic  Generalized Anxiety  Separation Anxiety | 2 (1.8-2.3)  2.2 (2.0-2.4)  2.0 (2.0-2.3)  2.3 (1.9-2.5)  2.0 (1.9-2.3)  1.9 (1.6-2.2)  1.8 (1.5-2.3)  46 (38-53)  45 (39-51)  48 (44-56)  43 (39-49)  46 (40-49)  47 (41-56)  46 (38-53)  48 (41-54) | 2.2 (1.8-2.5)  2.2 (2.0-2.7)  2.2 (2.1-2.4)  2.1 (1.8-2.5)  2.3 (2.0-2.7)  1.8 (1.6-2.1)  2.0 (1.5-2.5)  57 (45-64)  55 (44-65)  56 (45-67)  50 (43-62)  53 (46-62)  54 (48-68)  49 (43-56)  54 (45-64) | z = -1.0  z = -0.7  z = -1.7  z = 0.6  z = -2.0  z = 0.4  z = -0.8  z = -2.7**  z = -2.8**  z = -1.8  z = -2.2*  z = -3.2**  z = -2.5*  z = -1.4  z = -2.2* |
| **Scale** | **Controls**  (n=28) | **TS**  (n=38) | **Wilcoxon Rank Sum Test Statistic** |
| **Caregiver-Report Scales** |  |  |  |
| Family Assessment Device  Problem Solving  Communication  Roles  Affective Responsiveness  Affective Involvement  Behavior Control  General Functioning  PedsQL Family Impact Module  Physical Functioning  Emotional Functioning  Social Functioning  Cognitive Functioning  Communication  Worry  Daily Activities  Family Relationships  Total | 1.8 (1.7-2.1)  2.0 (1.7-2.2)  2.0 (1.8-2.2)  1.7 (1.3-2.1)  2.0 (1.9-2.2)  1.4 (1.2-1.8)  1.6 (1.4-2.0)  96 (75-100)  90 (65-100)  100 (72-100)  100 (83-100)  100 (83-100)  95 (75-100)  100 (71-100)  98 (63-100)  95 (67-99) | 1.8 (1.7-2.2)  1.8 (1.6-2.1)  2.1 (2.0-2.3)  1.7 (1.3-2.0)  2.0 (1.9-2.4)  1.6 (1.2-1.9)  1.7 (1.3-2.0)  77 (54-88)  60 (50-70)  85 (50-100)  75 (55-95)  67 (50-83)  55 (25-65)  67 (42-92)  70 (50-95)  73 (53-78) | z = -0.1  z = 1.3  z = -1.2  z = -0.1  z = -1.2  z = -0.6  z = -0.0  z = 2.9**  z = 4.0***  z = 2.2*  z = 3.2**  z = 3.7***  z = 4.9***  z = 3.4***  z = 2.9**  z = 3.9*** |
| Revised Children’s Anxiety and Depression Scale  Total Anxiety Depression  Total Anxiety  Total Depression  Obsessions-Compulsions  Social Phobia  Panic  Generalized Anxiety  Separation Anxiety | 53 (45-61)  52 (47-60)  53 (46-58)  45 (43-47)  55 (47-60)  51 (46-58)  56 (45-62)  50 (41-53) | 63 (55-70)  61 (52-68)  66 (54-77)  52 (45-65)  58 (51-65)  59 (53-71)  55 (47-64)  59 (50-78) | z = -3.0**  z = -2.6**  z = -3.2**  z = -2.8**  z = -1.5  z = -2.4*  z = -0.2  z = -3.0** |

*Note.*

* p < 0.05. ** p < 0.01. *** p < 0.001.

‡ Median (interquartile range)

**Table S5**

*Comparison of TS Group to Available Normative Samples or Clinical Threshold*

| **Scale** | **Normative or Clinical Threshold Score Citation** | **TS (n=38)** | **Normative or Clinical Threshold** | **Wilcoxon Signed Rank Test Statistic** |
| --- | --- | --- | --- | --- |
| **Adolescent-Report Scales** |  |  |  |  |
| Youth Quality of Life – Research Version  Sense of Self  Social Relationships  Environment  General Quality of Life  Total | Topolski et al. (2002) | 72.5^‡^ (55.7-81.4)  78.9 (68.6-90.7)  86 (71-93)  83.3 (60-100)  75.9 (63.2-90.3) | 78.8  80.8  87.6  86.9  82.2 | z = -3.2**  z = -1.0  z = -1.9  z = -2.4*  z = -2.1* |
| Self-Esteem Scale | Bagley et al. (2001) | 28.5 (25-34) | 31.26 | z = -1.9 |
| Daily Life Stressors Scale | Kearney et al. (1993) | 37 (27-51) | 33.21 | z = 1.6 |
| PROMIS Pediatric Peer Relationships Short Form | DeWalt et al. (2013) | 43.7 (38.8-50.9) | 50 | z = -3.0** |
| Family Assessment Device  Problem Solving  Communication  Roles  Affective Responsiveness  Affective Involvement  Behavior Control  General Functioning | Miller et al. (1985) | 2.2 (1.8-2.5)  2.2 (2.0-2.7)  2.2 (2.1-2.4)  2.1 (1.8-2.5)  2.3 (2.0-2.7)  1.8 (1.6-2.1)  2.0 (1.5-2.5) | 2.2  2.2  2.3  2.2  2.1  1.9  2.0 | z = -0.8  z = 1.0  z = -1.9  z = -0.6  z = 2.3*  z = -1.2  z = -0.2 |
| Revised Children’s Anxiety and Depression Scale  Total Anxiety Depression  Total Anxiety  Total Depression  Obsessions-Compulsions  Social Phobia  Panic  Generalized Anxiety  Separation Anxiety | Chorpita et al. (2000) | 57 (45-64)  55 (44-65)  56 (45-67)  50 (43-62)  53 (46-62)  54 (48-68)  49 (43-56)  54 (45-64) | 50 | z = 2.4*  z = 2.2*  z = 2.6**  z = 0.4  z = 2.1*  z = 2.8**  z = 0.0  z = 1.9 |
| **Scale** | **Normative or Clinical Threshold Score Citation** | **TS (n=38)** | **Normative or Clinical Threshold** | **Wilcoxon Signed Rank Test Statistic** |
| **Caregiver-Report Scales** |  |  |  |  |
| PROMIS Parent Peer Relationships Short Form | Varni et al. (2011) | 42.5 (36-51) | 50 | z = -3.1** |
| Family Assessment Device  Problem Solving  Communication  Roles  Affective Responsiveness  Affective Involvement  Behavior Control  General Functioning | Miller et al. (1985) | 1.8 (1.7-2.2)  1.8 (1.6-2.1)  2.1 (2.0-2.3)  1.7 (1.3-2.0)  2.0 (1.9-2.4)  1.6 (1.2-1.9)  1.7 (1.3-2.0) | 2.2  2.2  2.3  2.2  2.1  1.9  2.0 | z = -4.2***  z = -4.2***  z = -3.2**  z = -3.5***  z = -0.6  z = -4.4***  z = -3.1** |
| Revised Children’s Anxiety and Depression Scale  Total Anxiety Depression  Total Anxiety  Total Depression  Obsessions-Compulsions  Social Phobia  Panic  Generalized Anxiety  Separation Anxiety | Chorpita et al. (2000) | 63 (55-70)  61 (52-68)  66 (54-77)  52 (45-65)  58 (51-65)  59 (53-71)  55 (47-64)  59 (50-78) | 50 | z = 4.4***  z = 4.1***  z = 4.5***  z = 1.9  z = 3.6***  z = 4.0***  z = 2.6**  z = 3.2** |
| Conners-3 Parent Short QuikScore Form (T-scores)  Inattention  Hyperactivity / Impulsivity  Learning Problems  Executive Functioning  Aggression  Peer Relations | Conners (2008) | 71 (62-81)  81 (63-90)  57 (50-66)  61 (49-71)  51 (45-60)  59 (45-90) | 50 | z = 5.1***  z = 5.2***  z = 3.6***  z = 4.1***  z = 1.7  z = 3.8*** |

*Note.* The Perceived Stress Scale and the PedsQL Family Impact Module do not have clear normative data available.

* p < 0.05. ** p < 0.01. *** p < 0.001.

‡ Median (interquartile range)

**Table S6**

*Comparison of Control Group to Available Normative Samples or Clinical Threshold*

| **Scale** | **Control (n = 28)** | **Normative or Clinical Threshold** | **Wilcoxon Signed Rank Test Statistic** |
| --- | --- | --- | --- |
| **Adolescent-Report Scales** |  |  |  |
| Youth Quality of Life – Research Version  Sense of Self  Social Relationships  Environment  General Quality of Life  Total | 77.9 (63.2-86.4)  82.5 (73.6-88.6)  85.5 (77.5-91.5)  85 (68.3-95)  82.0 (71.4-90.1) | 78.8  80.8  87.6  86.9  82.2 | z = -1.2  z = -0.2  z = -1.6  z = -1.4  z = -0.6 |
| Self-Esteem Scale | 29.5 (26.5-34) | 31.26 | z = -1.2 |
| Daily Life Stressors Scale | 27.5 (18-37) | 33.21 | z = -1.6 |
| PROMIS Pediatric Peer Relationships Short Form 8a | 42.6 (38.8-47.4) | 50 | z = -3.4*** |
| Family Assessment Device  Problem Solving  Communication  Roles  Affective Responsiveness  Affective Involvement  Behavior Control  General Functioning | 2 (1.8-2.3)  2.2 (2.0-2.4)  2.0 (2.0-2.3)  2.3 (1.9-2.5)  2.0 (1.9-2.3)  1.9 (1.6-2.2)  1.8 (1.5-2.3) | 2.2  2.2  2.3  2.2  2.1  1.9  2.0 | z = -2.4*  z = 0.2  z = -2.9**  z = 0.2  z = -0.6  z = -0.5  z = 1.7 |
| Revised Children’s Anxiety and Depression Scale  Total Anxiety Depression  Total Anxiety  Total Depression  Obsessions-Compulsions  Social Phobia  Panic  Generalized Anxiety  Separation Anxiety | 46 (38-53)  45 (39-51)  48 (44-56)  43 (39-49)  46 (40-49)  47 (41-56)  46 (38-53)  48 (41-54) | 50 | z = -1.5  z = -2.0*  z = -0.1  z = -2.9**  z = -2.9**  z = -0.71  z = -1.7  z = -1.4 |
| **Scale** | **Control (n = 28)** | **Normative or Clinical Threshold** | **Wilcoxon Signed Rank Test Statistic** |
| **Caregiver-Report Scales** |  |  |  |
| PROMIS Parent Proxy Peer Relationships Short Form 7a (T-score) | 45 (42-51) | 50 | z = -2.4* |
| Family Assessment Device  Problem Solving  Communication  Roles  Affective Responsiveness  Affective Involvement  Behavior Control  General Functioning | 1.8 (1.7-2.1)  2.0 (1.7-2.2)  2.0 (1.8-2.2)  1.7 (1.3-2.1)  2.0 (1.9-2.2)  1.4 (1.2-1.8)  1.6 (1.4-2.0) | 2.2  2.2  2.3  2.2  2.1  1.9  2.0 | z = -3.4***  z = -3.1**  z = -3.6***  z = -3.7***  z = -2.1*  z = -4.4***  z = -3.3*** |
| Revised Children’s Anxiety and Depression Scale  Total Anxiety Depression  Total Anxiety  Total Depression  Obsessions-Compulsions  Social Phobia  Panic  Generalized Anxiety  Separation Anxiety | 53 (45-61)  52 (47-60)  53 (46-58)  45 (43-47)  55 (47-60)  51 (46-58)  56 (45-62)  50 (41-53) | 50 | z = 1.4  z = 1.2  z = 1.6  z = -2.3*  z = 1.8  z = 1.0  z = 2.4*  z = -0.8 |
| Conners-3 Parent Short QuikScore Form (T-scores)  Inattention  Hyperactivity / Impulsivity  Learning Problems  Executive Functioning  Aggression  Peer Relations | 57 (49-69)  57 (48-64)  49 (46-54)  53 (47-62)  49 (45-52)  53 (46-68) | 50 | z = 3.0**  z = 3.0**  z = -0.3  z = 1.9  z = -1.1  z = 2.0* |

*Note.* The Perceived Stress Scale and the PedsQL Family Impact Module do not have clear normative data available.

* p < 0.05. ** p < 0.01. *** p < 0.001

**Table S7**

*Additional* *Details of* *LASSO Regression Analysis for TS Participants^†^*

| **Imputation Dataset Number** | **Number of Control Variables Selected** | **Wald 𝝌^2^(3)** | **β^‡^ for Daily Life Stressor Scale** | **β for PROMIS Pediatric Peer Relationship SF 8a Raw Score** | **β for Family Assessment Device – General Functioning Raw Score** |
| --- | --- | --- | --- | --- | --- |
| 1 | 5 | 27.0**** | -0.025 | 0.22 | -8.6**** |
| 2 | 6 | 25.3**** | 0.034 | 0.25 | -8.4**** |
| 3 | 5 | 26.8**** | -0.014 | 0.23 | -8.6**** |
| 4 | 5 | 27.3**** | -0.029 | 0.22 | -8.6**** |
| 5 | 6 | 25.4**** | 0.027 | 0.25 | -8.4**** |
| 6 | 5 | 27.0**** | -0.025 | 0.22 | -8.6**** |
| 7 | 5 | 27.0**** | -0.025 | 0.22 | -8.6**** |
| 8 | 5 | 27.0**** | -0.025 | 0.22 | -8.6**** |
| 9 | 6 | 25.3**** | 0.032 | 0.25 | -8.4**** |
| 10 | 6 | 26.0**** | 0.009 | 0.24 | -8.5**** |
| 11 | 5 | 27.0**** | -0.025 | 0.22 | -8.6**** |
| 12 | 5 | 27.0**** | -0.025 | 0.22 | -8.6**** |
| 13 | 6 | 25.4**** | 0.029 | 0.25 | -8.4**** |
| 14 | 5 | 27.0**** | -0.025 | 0.22 | -8.6**** |
| 15 | 5 | 27.3**** | -0.029 | 0.22 | -8.6**** |
| 16 | 5 | 27.3**** | -0.029 | 0.22 | -8.6**** |
| 17 | 5 | 27.0**** | -0.025 | 0.22 | -8.6**** |
| 18 | 6 | 25.9**** | 0.009 | 0.24 | -8.5**** |
| 19 | 6 | 26.0**** | 0.010 | 0.24 | -8.5**** |
| 20 | 6 | 25.5**** | 0.025 | 0.25 | -8.4**** |
| Mean (SD) | 5.4 (0.5) | 25.3-27.3^^^ | -0.0065 (0.025) | 0.23 (0.013) | -8.5 (0.091) |

*Note.* ^†^ For LASSO regression of each imputed dataset for TS participants, YQOL-R served as the dependent variable; Daily Life Stressors Scale score, PROMIS Pediatric Peer Relationships Short Form 8a raw score, and adolescent-reported Family Assessment Device – General Functioning score served as independent variables of interest; and the following served as control variables: age, sex, YGTSS Total Tic Score, Self-Esteem Scale score, Perceived Stress Scale score, adolescent-reported RCADS Total Anxiety-Depression raw score, and Conners-3 Parent Short QuikScore Form raw scores for all subscales (Inattention, Hyperactivity/Impulsivity, Learning Problems, Executive Functioning, Aggression, Peer Relations, Positive Interpretations, and Negative Interpretations).

‡ Coefficients are unstandardized

^ range

* p < 0.05. *** p< 0.001; **** p < 0.0001; SD = standard deviation

**Table S8**

*Additional* *Details of* *LASSO Regression Analysis for Control Participants^†^*

| **Imputation Dataset Number** | **Number of Control Variables Selected** | **Wald 𝝌^2^(3)** | **β for Daily Life Stressor Scale** | **β for PROMIS Pediatric Peer Relationship SF 8a Raw Score** | **β for Family Assessment Device – General Functioning Raw Score** |
| --- | --- | --- | --- | --- | --- |
| N/A | 5 | 41.5**** | 0.21 | 0.24 | -15.0*** |

*Note.* ^†^ For LASSO regression of control participants, YQOL-R served as the dependent variable; Daily Life Stressors Scale score, PROMIS Pediatric Peer Relationships Short Form 8a raw score, and adolescent-reported Family Assessment Device – General Functioning score served as independent variables of interest; and the following served as control variables: age, sex, Self-Esteem Scale score, Perceived Stress Scale score, adolescent-reported RCADS Total Anxiety-Depression raw score, and Conners-3 Parent Short QuikScore Form raw scores for all subscales (Inattention, Hyperactivity/Impulsivity, Learning Problems, Executive Functioning, Aggression, Peer Relations, Positive Interpretations, and Negative Interpretations).

* p < 0.05; *** p< 0.001; **** p < 0.0001

**Figure S1**

*Scatterplots of Select Scale Scores Versus YQOL-R Total Score*

*Note.* Higher YQOL-R total score indicates higher quality of life. Higher Daily Life Stressors Scale score indicates greater perceived burden from daily life stressors; higher PROMIS Pediatric Peer Relationships Short Form 8a raw score indicates greater friendship quality and peer acceptance; higher Family Assessment Device – General Functioning Scale score indicates unhealthier family functioning; and higher YGTSS Total Tic Score indicates greater tic severity.

**Figure S2**

*Scale Correlation Matrix for Control Participants*


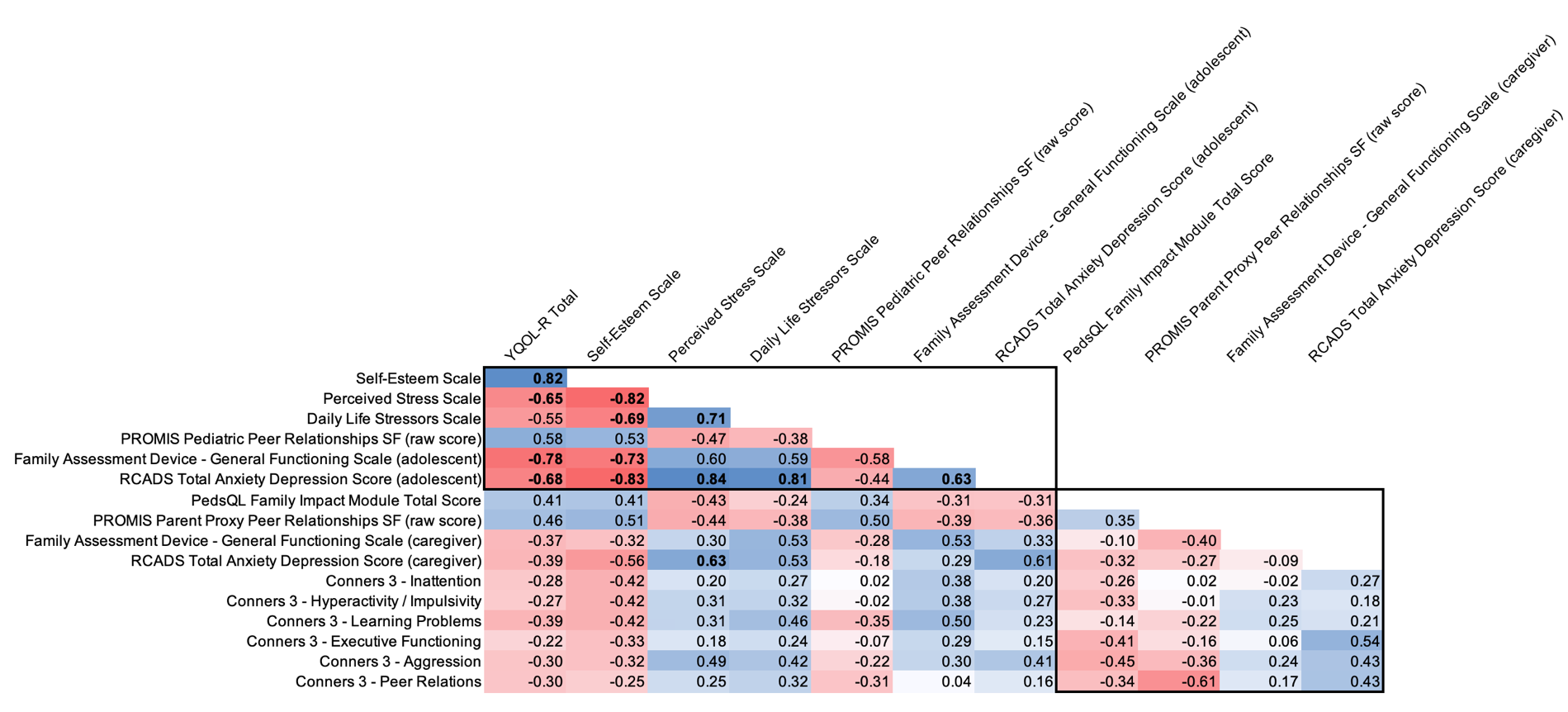


*Note.* The bold-outlined section in the upper left quadrant of the matrix contains correlations between adolescent-reported measures. The bold-outlined section in the lower right quadrant of the matrix contains correlations between caregiver-reported measures. Red and blue shading signify negative and positive correlations, respectively, while intensity of shading signifies the strength of the correlation. Bolded values indicate statistically significant correlation following correction for multiple comparisons.

**References**

Bagley, C., & Mallick, K. (2001). Normative data and mental health construct validity for the Rosenberg Self-Esteem Scale in British adolescents. *International Journal of Adolescence and Youth, 9*(2-3), 117-126.

Chorpita, B.F., Yim, L.M., Moffitt, C.E., Umemoto, L.A., & Francis, S.E. (2000). Assessment of symptoms of DSM-IV anxiety and depression in children: A revised child anxiety and depression scale. *Behaviour Research and Therapy, 38,* 835-955.

Conners, K.C. (2008). Conners 3^rd^ edition. Toronto, Ontario, Canada: Multi-Health Systems.

DeWalt, D., Thissen, D., Stucky, B., Langer, M., Morgan Dewitt, E., Lai, J…Varni, J. (2013). PROMIS Pediatric Peer Relationships Scale: Development of a peer relationships item bank as part of social health measurement. *Health Psychology. 32*(10), 1093-1103.

Kearney, C., Drabman, R., & Beasley, J.F. (1993). The trials of childhood: The development, reliability, and validity of the daily life stressors scale. *Journal of Child and Family Studies, 2,* 371-388.

Miller, I., Epstein, N., Bishop, D., & Keitner, G. (1985). The McMaster Family Assessment Device: Reliability and validity. *Journal of Marital and Family Therapy, 11*(4), 345-356. https://doi.org/10/1111/j.1752-0606.1985.tb00028.x

Topolski, T. D., Edwards, T. C., and Patrick .D L. (2002). User's manual and interpretation guide for the Youth Quality of Life (YQOL) Instruments. Seattle, WA: University of Washington, Dept. of Health Services

Varni, J.W., Thissen, D., Stucky, B.D., Liu, Y. Gorder, H., Irwin, D.E., DeWitt, E.M., Lai, J., Amtmann, D., & DeWalt, D.A. (2011). PROMIS parent proxy report scales: An item response theory analysis of the parent proxy report item banks. *Quality of Life Research, 21,* 1223-1240.
